# Study Profile of the Tohoku Medical Megabank Community-Based Cohort Study

**DOI:** 10.2188/jea.JE20190271

**Published:** 2021-01-05

**Authors:** Atsushi Hozawa, Kozo Tanno, Naoki Nakaya, Tomohiro Nakamura, Naho Tsuchiya, Takumi Hirata, Akira Narita, Mana Kogure, Kotaro Nochioka, Ryohei Sasaki, Nobuyuki Takanashi, Kotaro Otsuka, Kiyomi Sakata, Shinichi Kuriyama, Masahiro Kikuya, Osamu Tanabe, Junichi Sugawara, Kichiya Suzuki, Yoichi Suzuki, Eiichi N Kodama, Nobuo Fuse, Hideyasu Kiyomoto, Hiroaki Tomita, Akira Uruno, Yohei Hamanaka, Hirohito Metoki, Mami Ishikuro, Taku Obara, Tomoko Kobayashi, Kazuyuki Kitatani, Takako Takai-Igarashi, Soichi Ogishima, Mamoru Satoh, Hideki Ohmomo, Akito Tsuboi, Shinichi Egawa, Tadashi Ishii, Kiyoshi Ito, Sadayoshi Ito, Yasuyuki Taki, Naoko Minegishi, Naoto Ishii, Masao Nagasaki, Kazuhiko Igarashi, Seizo Koshiba, Ritsuko Shimizu, Gen Tamiya, Keiko Nakayama, Hozumi Motohashi, Jun Yasuda, Atsushi Shimizu, Tsuyoshi Hachiya, Yuh Shiwa, Teiji Tominaga, Hiroshi Tanaka, Kotaro Oyama, Ryoichi Tanaka, Hiroshi Kawame, Akimune Fukushima, Yasushi Ishigaki, Tomoharu Tokutomi, Noriko Osumi, Tadao Kobayashi, Fuji Nagami, Hiroaki Hashizume, Tomohiko Arai, Yoshio Kawaguchi, Shinichi Higuchi, Masaki Sakaida, Ryujin Endo, Satoshi Nishizuka, Ichiro Tsuji, Jiro Hitomi, Motoyuki Nakamura, Kuniaki Ogasawara, Nobuo Yaegashi, Kengo Kinoshita, Shigeo Kure, Akio Sakai, Seiichiro Kobayashi, Kenji Sobue, Makoto Sasaki, Masayuki Yamamoto

**Affiliations:** 1 Tohoku Medical Megabank Organization, Tohoku University, Sendai, Japan; 2 Graduate School of Medicine, Tohoku University, Sendai, Japan; 3 Iwate Tohoku Medical Megabank Organization, Disaster Reconstruction Center, Iwate Medical University, Iwate, Japan; 4 School of Medicine, Iwate Medical University, Morioka, Japan; 5 Saitama Prefectural University, Saitama, Japan; 6 Tohoku University Hospital, Tohoku University, Sendai, Japan; 7 International Research Institute of Disaster Science, Tohoku University, Sendai, Japan; 8 Teikyo University School of Medicine, Tokyo, Japan; 9 Radiation Effects Research Foundation, Hiroshima, Japan; 10 Ageo Central General Hospital, Saitama, Japan; 11 Faculty of Medicine, Tohoku Medical and Pharmaceutical University, Sendai, Japan; 12 Setsunan University, Osaka, Japan; 13 Institute for Biomedical Sciences, Iwate Medical University, Iwate, Japan; 14 Graduate School of Dentistry, Tohoku University, Sendai, Japan; 15 Institute of Development, Aging and Cancer, Tohoku University, Sendai, Japan; 16 Graduate School of Information Sciences, Tohoku University, Sendai, Japan; 17 Kyoto University Graduate School of Medicine Faculty of Medicine, Kyoto, Japan; 18 Center for Advanced Intelligence Project, RIKEN, Tokyo, Japan; 19 Miyagi Cancer Center, Miyagi, Japan; 20 Tokyo Medical and Dental University, Tokyo, Japan; 21 The JIKEI University School of Medicine, Tokyo, Japan; 22 Iwate Medical University School of Nursing, Iwate, Japan; 23 Iwate Medical University, Morioka, Japan

**Keywords:** prospective cohort studies, Great East Japan Earthquake, genome cohort

## Abstract

**Background:**

We established a community-based cohort study to assess the long-term impact of the Great East Japan Earthquake on disaster victims and gene-environment interactions on the incidence of major diseases, such as cancer and cardiovascular diseases.

**Methods:**

We asked participants to join our cohort in the health check-up settings and assessment center based settings. Inclusion criteria were aged 20 years or over and living in Miyagi or Iwate Prefecture. We obtained information on lifestyle, effect of disaster, blood, and urine information (Type 1 survey), and some detailed measurements (Type 2 survey), such as carotid echography and calcaneal ultrasound bone mineral density. All participants agreed to measure genome information and to distribute their information widely.

**Results:**

As a result, 87,865 gave their informed consent to join our study. Participation rate at health check-up site was about 70%. The participants in the Type 1 survey were more likely to have psychological distress than those in the Type 2 survey, and women were more likely to have psychological distress than men. Additionally, coastal residents were more likely to have higher degrees of psychological distress than inland residents, regardless of sex.

**Conclusion:**

This cohort comprised a large sample size and it contains information on the natural disaster, genome information, and metabolome information. This cohort also had several detailed measurements. Using this cohort enabled us to clarify the long-term effect of the disaster and also to establish personalized prevention based on genome, metabolome, and other omics information.

## INTRODUCTION

On the afternoon of March 11, 2011, the Great East Japan Earthquake (GEJE) occurred in a large area within eastern Japan, and the following tsunami devastated coastal areas of northern Japan. Consequently, 15,897 individuals were confirmed dead, and 2,534 were reported missing in Japan. In Miyagi and Iwate Prefectures, 14,216 deaths and 2,334 missing people were reported.^1^ Thus, Miyagi and Iwate Prefectures could be considered most severely impacted by GEJE, with the most damage.

There is a pressing concern regarding increased neuropsychiatric disorders, such as posttraumatic stress disorder (PTSD),^2^ cigarette and alcohol consumption caused by psychological stress,^3^ and high blood pressure (BP)^4^ and accompanying cardiovascular diseases (CVD)^5^ due to disaster-related stress in the areas where people were greatly affected by the GEJE. Additionally, some patients with high BP, diabetes, or other diseases may have had to discontinue treatment owing to psychosocial adverse effects.^6^ If such conditions are not addressed, the frequency of cerebrovascular diseases, CVD, cancer, or suicide might subsequently increase. Thus, it is necessary to clarify the associations between the multidimensional factors and the risk of short-term onset diseases (mental distress, hypertension, and infectious diseases), as well as to identify high-risk subjects and provide support to the individuals and the community.

In addition to environmental and lifestyle factors, genetic factors might affect the disease onset or disease progression. It has been clarified that genetic factors contribute to the development of risk factors, such as elevated body mass index (BMI), high BP, and common diseases, such as stroke, CVD, cancer, and mental disorders. Furthermore, lifestyle habits, such as alcohol consumption, are modified by genetic factors,^7^^,^^8^ so there is a need for studies that investigate the impact of genetic factors on relation between lifestyle and diseases (ie, gene-environment interactions). Thus, we decided to ask participants to provide their genetic information.

To overcome the above-mentioned issues, we established the Tohoku Medical Megabank (TMM) Project, which is conducted by Tohoku University Tohoku Medical Megabank Organization (ToMMo) and Iwate Medical University Iwate Tohoku Medical Megabank Organization (IMM).^9^ We decided to conduct the TMM Project in Miyagi and Iwate Prefectures and started two prospective cohort studies in Miyagi and Iwate Prefectures: a population-based adult cohort study, the TMM Community-Based Cohort Study (TMM CommCohort Study), which recruited more than 80,000 participants, and a birth and three-generation cohort study, the TMM Birth and Three-Generation Cohort Study (TMM BirThree Cohort Study), which recruited more than 70,000 participants, including fetuses and their parents, siblings, grandparents, and extended family members.^10^

The objective of TMM CommCohort Study is to assess the long-term impact of the GEJE on disaster victims. We will conduct long-term follow-up analyses to investigate the cause-specific mortality and incidences of cancer, cerebrovascular diseases, and heart diseases as the main outcomes, as well as the risk factors of these diseases. Additionally, we also assess the gene-environment interactions on the incidence of the above-mentioned major diseases. The associations between genetic risk scores and the main outcomes—particularly the absolute risk assessment—and the associations between genetic risk scores combined with environmental factors and the main outcomes—particularly the development of new disease risk charts, such as the Framingham risk score,^11^ a risk prediction model developed by the national cancer center^12^^,^^13^; NIPPON DATA risk chart^14^; and Suita score^15^—through the assessment of absolute risks will be conducted.

## METHODS

### Recruitment

#### Design, inclusion criteria, and exclusion criteria

TMM CommCohort Study is a community-based prospective cohort study conducted from 2013 in the areas affected by the GEJE. Inclusion criteria were persons aged 20 years old or over who were registered in the basic resident register of all municipalities in Miyagi Prefecture and Iwate Prefecture at the time of enrollment. Despite the above criteria, we did not ask participants aged 75 years and older at the site of municipality based health check-up. Individuals were excluded from the TMM CommCohort Study if they did not consent to participate in the study and/or if they were not able to complete the study questionnaires.

#### Ethical Committee and Genome Cohort Cooperation Working Group

The TMM CommCohort Study was approved by Ethical Committee of ToMMo (the first approval: 2012-4-617 and latest approval: 2018-4-087) and the Medical Ethics Committee of Iwate Medical University (HG H25-2). The study protocol was compiled with inputs from the Genome Cohort Collaboration Promotion Working Group, which consisted of leaders of the genome cohort in Japan. All participants provided informed consent to the TMM Project to have their data from the specific health check-ups and on medical expenditure utilized, or several other official sources of information for the sake of this study.

#### Recruitment

**Figure 1.**
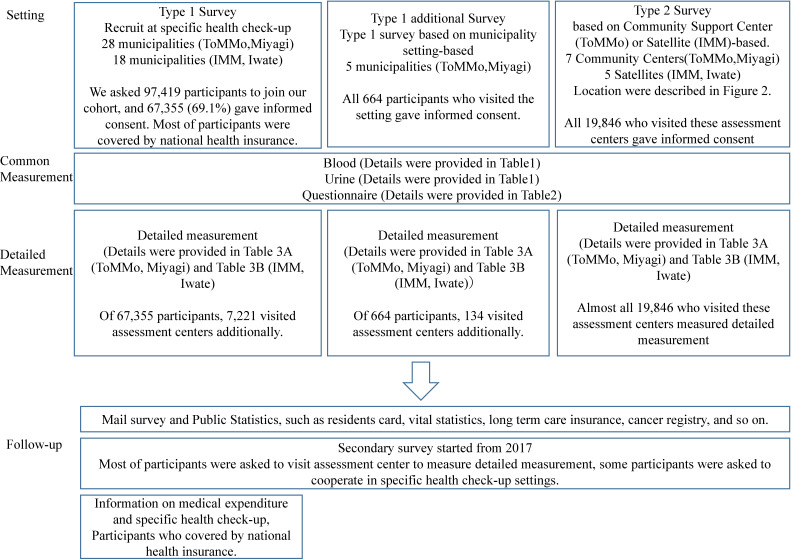
Outlines of Tohoku Medical Megabank Community based Cohort Study 2013–2015 (fiscal years)

In the TMM CommCohort Study, we recruited potential participants through two major approaches (Figure 1). The Type 1 survey collected basic information, such as blood and urine, questionnaire, and municipal health check-up data (eg, height, weight, and blood pressure). The Type 2 survey was conducted at the TMM facility without going through the municipal health check-ups with detailed measurement in addition to measurement list of the Type 1 survey.

#### Type 1 survey based on municipal health check-up

The first type of survey was performed in specific municipal health check-up sites. We visited the sites and ask participants to join our cohort. All participants living in Japan were eligible to undergo this screening, which is provided by an insurer. These check-ups focused on visceral fat obesity, which is used to screen for individuals with risk factors of CVD, such as hypertension, diabetes, dyslipidemia, and metabolic syndrome. Participants who attend the health check-up conducted by the municipality were mainly covered members of the National Health Insurance (NHI).^16^

In the health check-up settings, researchers explained the TMM Project overview and cohort study contents to participants. The briefings were to be performed mainly by staff, instructors, and Genome Medical Research Coordinators (GMRCs) who belonged to the ToMMo and IMM. While participants were waiting for health checkups and before blood was drawn, informed consent was obtained with face-to-face individual interviews by trained GMRC, and each participant checked all the items on the written form and signed. When they consented to join our cohort, additional blood was drawn from participants and they were handed questionnaires, which they would complete in their house and would send back to our research center.

#### Type 1 survey based on municipality setting-based (Type 1 Additional) survey

When there was a limitation with the setting of municipal specific health check-ups (the size of the venue), we conducted the Type 1 Additional survey. The survey was conducted in ToMMo only and was conducted on different dates from those of the specific health check-ups in the municipality; the municipality and the ToMMo arranged for the survey to be conducted in a selected place at a specified date. The way for obtaining informed consent was similar to the Type 1 survey.

#### Type 2 survey based on assessment center-based (Type 2) survey

The Type 2 survey was performed in an assessment center, the Community Support Center in ToMMo and Satellite in IMM. All eligible participants (ie, residents in Miyagi and Iwate prefecture aged 20 years and over) could participate in the Type 2 survey, and participants were administered several detailed measurements.

TMM Project conducted advertisements for enrollment using mass media, such as newspaper fliers, webpages, television and radio, distribution of advertisement leaflets to public institutions, and advertisement at the specific health check-up venues. For those who wished to participate in the study, the assessment centers office arranged appointments for them to visit on specified dates. The TMM Project outline and cohort study details were explained to examinees at the survey (briefing). Informed consent was obtained in writing from each participant by the GMRC. This survey collected information on physiological measurements, in addition to those collected for the Type 1 and Type 1 Additional survey.

**Figure 2.**
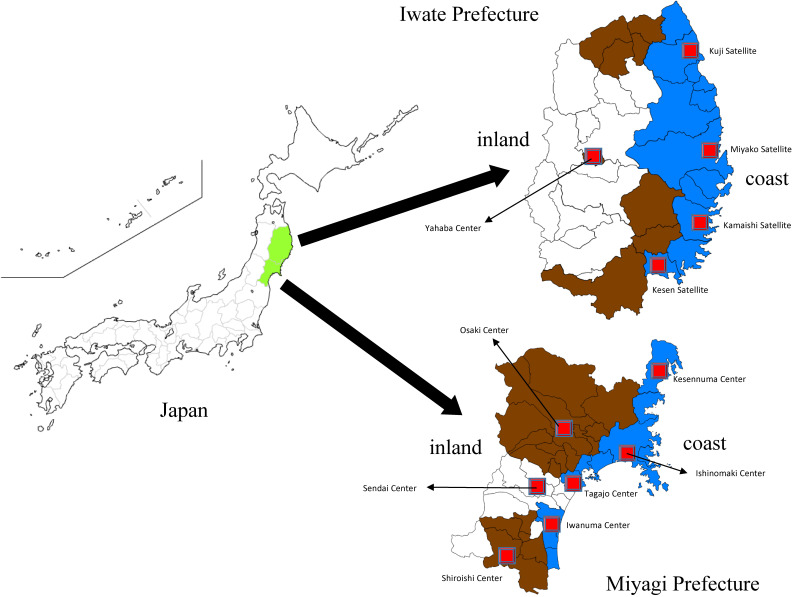
Location of Miyagi prefecture, Iwate prefecture, Japan, location of survey implementation municipalities, and Community Support Center (ToMMo, Miyagi) and Satellite (IMM, Iwate)

The municipalities that cooperated with the Type 1 or Type 1 Additional survey are provided in Figure 2. In Figure 2, we also describe the location of assessment centers that performed the Type 2 surveys.

### Measurements

#### Blood and urine collection and testing

For all of the surveys using different recruitment methods, six tubes of blood were collected. At the Type 1 survey, participants were asked to provide extra blood in addition to that required for the specific health check-ups.

The common blood collection amount in ToMMo and IMM were 34 mL. The measured blood consisted of 7 mL in an EDTA2Na blood tube, 5 mL in a sodium heparin blood tube, and two serum blood tubes containing 9 mL of blood, completing blood count tests (2 mL), and for a blood sugar or HbA1c test (2 mL).

For urine collection, at the Type 1 survey, urine samples that were leftover following measurement for the specific health checkups were stored in urine centrifuge tubes. For the Type 1 Additional surveys or the Type 2 survey in ToMMo, participants were requested to collect urine at home on the morning of the survey; thereafter, they were requested to bring the urine to the sites or assessment centers. For Type 2 surveys, a spot urine sample was collected at each of the satellites in IMM. Details of blood and urine tests conducted for study purposes are provided in Table 1.

**Table 1.** Details of blood and urine tests at baseline for the TMM CommCohort study from 2013–2015 (fiscal years)

Measurement	Measurement lists	Details of the measurement methods	Fiscal years	

			2013	2014–2015
Blood	N-terminal pro–B-type natriuretic peptide (NT-proBNP)	ECLIA (electro-chemiluminescence immunoassay)	○	IMM only
	Glycoalbumin (GA)	Enzymatic method	○	IMM only
	Creatinine (CRE)	Enzymatic method	○	○
	Allergen-Specific immunoglobulin E (IgE) antibody test	ELISA (enzyme-linked immunosorbent assay)	33-item	5-item
	Helicobacter pylori antibody test	EIA (enzyme immunoassay)	○	○
	Serum pepsinogen (PG) test	CLEIA (chemiluminescent enzyme immunoassay)	○	○
	Aspartate aminotransferase (AST)	JSCC transferable method	○	○
	Alanine aminotransferase (ALT)	JSCC transferable method	○	○
	Gamma-glutamyl transferase (GGT)	JSCC transferable method	○	○
	Urea nitrogen (UN)	Urease-leucine dehydrogenase/Ultraviolet absorption spectrophotometry	○	○
	Uric acid (UA)	Enzymatic method (Uricase-POD)	○	○
	Glucose	Enzymatic UV test (hexokinase method)	○	○
	Triglycerides (TG)	Enzymatic method	○	○
	Total cholesterol (T-Cho)	Ultra. Violet-End (UV-End) method using cholesterol dehydrogenase (CDH)	○	○
	Low-density lipoprotein cholesterol (LDL-C)	Friedwald formula to calculate it in Miyagi/Direct method in Iwate	○	○
	High-density lipoprotein cholesterol (HDL-C)	Direct method	○	○
	Complete blood count (CBC)	Seath flow electrical resistance method/Sodium lauryl sulfate (SLS) hemoglobin method/Flow cytometry	○	○
	Complete blood count (CBC) with differential	May-Grunwald-Giemsa staining method	○	○
	Hemoglobin A1c (HbA1c)	Latex agglutination turbidimetry in Miyagi/High performance liquid chromatography (HPLC) in Iwate	○	○
	Immunoglobulin E (IgE) test	Fluorescent enzyme immunoassays	○	○
	Cystatin C	Latex agglutination turbidimetry method	○	○
	High-sensitivity cardiac troponin T	ECLIA (electro-chemiluminescence immunoassay)	IMM only	IMM only

Urine	Microalbumin	Quantitative immunoturbidimetry	○	○
	Creatinine	Enzymatic method	○	○
	Sodium	Quantitative ion selective electrodes (ISE)	○	○
	Chloride	Quantitative ion selective electrodes (ISE)	○	○
	Potassium	Quantitative ion selective electrodes (ISE)	○	○

○ ToMMo, Miyagi Prefecture; IMM, Iwate Prefecture.

#### Questionnaires

For the Type 1 surveys, after participants provided informed consent, they were handed questionnaires for self-administration. Staff asked participants to complete and return them in person within the 2 weeks following survey administration. For the Type 2 surveys, following the organization of appointments for the participants to visit the assessment centers, participants were sent questionnaires via mail.

The following question items were established on the basis of the question items from the Japan Multi-Institutional Collaborative Cohort Study^17^ (J-MICC Study), a cohort study consisting of tens of thousands of the general population, and based on the results of the Japan Public Health Center-based Prospective Study^18^ (JPHC Study). The J-MICC study intended to investigate the influence of both living circumstances and genetic contributors as risk factors of cancer and other lifestyle-related diseases. Such information would be useful in gaining information to establish life-style related disease prevention methods suitable for individual constitutions, or tailor-made prevention. The JPHC Study was conducted as a joint study by a designated group to establish evidence on the benefit of health maintenance and improvements including cancer prevention based on multipurpose cohort studies. Additionally, we have included items from the survey performed in the GEJE-affected area, such as K6^19^ and Lubben Social Network Scale-6 (LSNS-6),^20^ since our cohort assessed victims of GEJE.^21^ Moreover, the questionnaire included unique items regarding the genetic disposition and birthplace of family members. A detailed list of the items included in the questionnaires is shown in Table 2. In IMM (Iwate), the participants were also asked to complete additional questionnaires.

**Table 2.** Details of the questionnaires at baseline for the TMM CommCohort study from 2013–2015 (fiscal years)

Measurement	Measurement lists	ToMMo and IMM
Basic information	Sex, age in years at baseline, height, weight, weight at 20 years or age, educational background, and the degree of housing damage by the Great East Japan Earthquake (GEJE)	○
Physical activities	Presence of busier season involving higher physical activity than usual in the past year	○
	Frequency of physical activities and hours spent per session in the past year (commuting, work, and household chores)	
	Frequency of physical activities and hours spent per session in the past year (leisure time)	
Alcohol drinking	Drinking status	○
Cigarette smoking	Smoking status and information on passive smoking	○
Psychological stress	Self-reported stress, Kessler Psychological Distress Scale (K6)	○
Medications, dietary supplements, and health foods	Information on drugs and regularly used supplements	○
Family structure and pet breeding	Marital status, numbers of family members, cohabitation status, and pet breeding (dogs, cats, and others)	○
Health related information	Self-rated health, treatment status of hypertension, diabetes, or hyperlipidemia, and dental treatment, and past history of diseases	○
Genetic predisposition	Type of earwax and flushing response by alcohol intake	○
Occupation	Employment status, classification of industries and occupation	○
Sleep	Hours of sleep, Athens Insomnia Scale (AIS), and information on sleep medications	○
Social network	Lubben social network scale (LSNS-6) and social capital	○
Depressive symptoms	Center for epidemiologic studies depression scale (CES-D)	○
Memory and damage of the GEJE	Posttraumatic stress reaction due to the GEJE	○
Information on obstetrics and gynecology (women only)	Age at menarche, and age at first pregnancy	○
Dietary information	Food frequency questionnaire (FFQ), and several diet related questions	○
Life events	Holmes and Rahe stress scale	IMM only
Trait of worry	Penn State Worry Questionnaire (PSWQ)	IMM only
Problematic alcohol use	CAGE	IMM only

○ ToMMo, Miyagi Prefecture; IMM, Iwate Prefecture.

#### Additional testing at Type 2 survey

In order to perform a more detailed assessment of the physiological health status of the participants, several additional physiological measurements were conducted. Physiological measurements take about 2 hours. In ToMMo, ophthalmic examinations, hearing acuity, respiratory function tests, estimated central BP, calcaneal ultrasound bone mineral density, muscular strength, oral examinations, and additional questions were asked using tablet personalized computers.

Additionally, for participants who agreed to have measurements taken at home, we asked them to measure home blood pressure and step counts for 2 weeks. In IMM, ophthalmic examinations, calcaneal ultrasound bone mineral density, visceral fat, electrocardiography, pulse wave velocity, carotid echography, and flow mediated dilation tests were performed. We describe the list of physiological measurements taken and the devices used at the assessment centers in Table 3A and Table 3B. As shown in Figure 1, some participants who were recruited in the Type 1 survey visited the assessment centers later and received additional testing.

**Table 3A.** Additional testing at Type 2 survey based on Community Support Center (ToMMo)

Measurements	Company	Model number
Height	A&D Company, Limited	AD-6400
Weight and body composition	Biospace Company, Limited	InBody720
Waist circumference	—	—
Touchscreen questionnaire	Fujitsu, Limited	STYLISTIC Q572/F
Axial length	Tomey Corporation	OA-1000
Intraocular pressure	Canon Lifecare Solutions, Incorporation	TX-20P
Optical coherence tomography	Topcon, Corporation	3D OCT-2000
Color retinal photography	Canon Lifecare Solutions, Incorporation	CR-2 PLUS
Refraction and keratometry	Canon Lifecare Solutions, Incorporation	RK-F2
Hearing acuity	Rion, Company, Limited	AA-H1
Respiratory function	Chest M.I., Incorporation	HI-801
Respiratory impedance	Chest M.I., Incorporation	MostGraph-01
Estimated central aortic blood pressure, casual blood pressure and heart rate	Omron Healthcare, Company, Limited	HEM-9000AI
Carotid ultrasound imaging	Panasonic Healthcare, Company, Limited	GM-72P00A
Calcaneal ultrasound bone mineral density	Ishikawa Seisakusho, Limited	UBM-3000
Leg extension strength	Takei Scientific Instruments, Company, Limited	T.K.K.1865
Grip strength	Tsutsumi, Company, Limited	YD(110kg)
Oral examination	—	—
Oral bacteriological examination	—	—
Home blood pressure^*^	Omron Healthcare, Company, Limited	HEM-7080IC
Number of steps per day^*^	Omron Healthcare, Company, Limited	HJ-205IT

^*^Measurement for 2 weeks.

ToMMo, Miyagi Prefecture.

**Table 3B.** Additional testing at Type 2 survey based on Satellite (IMM)

Measurements	Company	Model number
Height and weight	A&D Company, Limited	AD-6350
Waist circumference	—	—
Blood pressure and heart rate	Omron Healthcare, Company, Limited	HBP-T105S-N
Calcaneal ultrasound bone mineral density	Ishikawa Seisakusho, Limited	UBM-3000
Visceral fat by bioelectrical impedance analysis	Omron Healthcare, Company, Limited	HDS-2000
Electrocardiogram	Fukuda Denshi, Company, Limited	FCP-8321
Pulse wave velocity	Omron Colin, Company, Limited	BP-203RPEIII
Carotid ultrasound imaging	Panasonic Healthcare Company, Limited	GM-72P00A
Flow mediated dilation	UNEX, Corporation	UNEXEF 38G
Color retinal photography	NIDEK, Company, Limited	AFC-330
Optical coherence tomography	NIDEK, Company, Limited	RS-3000 Advance
Axial length	NIDEK, Company, Limited	AL-Scan

IMM, Iwate Prefecture.

#### Data feedback

We returned the results of blood, urine, questionnaire, and other measurements to participants within 2 to 6 months. If they showed extraordinary data, which might affect the health immediately, we returned the data as soon as possible. We also provided mental health follow-up when the participants showed high risk for depression or post-traumatic stress response.

### Follow-up test

We performed several ways to ascertain follow-up information of our participants. In brief, the ways were mail survey; information on medical expenditure and health check-up; medical record review; public statistics, such as basic resident registration and vital statistics; and the following secondary surveys.

#### Mail survey

We sent surveys that collected information on self-reported onset of diseases; change in psychological distress, assessed by K6; and treatment status of hypertension, diabetes, and hyperlipidemia via postal mail. Additionally, the surveys included questions regarding the onset of the following diseases: cerebrovascular diseases, myocardial infarction (MI), angina pectoris, chronic obstructive pulmonary disease (COPD), endoscopic treatment and/or surgery owing to gastric ulcers or duodenal ulcers, influenza, PTSD, bronchial asthma, atopic dermatitis, and glaucoma.

#### Information on medical expenditure and health check-ups

To ascertain information on the specific health check-ups and medical expenditures, we asked the municipality to provide data on blood and urine tests conducted during the specific health check-ups for participants covered by the NHI. The NHI covers almost all medical treatment required for beneficiaries, including diagnostic tests, medication, surgery, supplies and materials, physician and other staffing costs, and most dental treatment. NHI data are reported separately by inpatient and outpatient files. When a beneficiary is withdrawn from the NHI owing to death or emigration, the corresponding reasons and data are entered on the NHI withdrawal history files. Both NHI claims and withdrawal history files were linked with our baseline survey data files using the beneficiary ID number. We also asked the municipality to provide information on medical expenditure withdrawn from the NHI. This information enabled us to clarify the participants who were admitted to hospital.

#### Medical record review

Information on the self-reported onset of diseases and hospital admissions were ascertained using data from the mail surveys or from the medical expenditure reports from the NHI. However, it is well known that self-reported onset of diseases has a lower positive predictive value (PPV). In the JPHC Study, the PPVs for self-reported cerebrovascular diseases and MI were 57% and 43%, respectively.^22^ Therefore, we confirmed our results by conducting medical record examinations.

Conversely, the sensitivity of a survey regarding the development of cerebrovascular diseases and MI was 70% and 82%, respectively.^22^ Thus, they confirmed that self-reported onset of cerebrovascular diseases and MI was sensitive enough to screen for baseline cerebrovascular diseases and MI in Japanese cohort studies. In addition to chart reviews, we collected information on disease registration, such as cancer or CVD (currently Iwate Prefecture only).

#### Public statistics

In principle, information on the address of the subjects was confirmed using the follow-up mail surveys sent by participants. If a participant did not return the follow-up mail survey or the TMM Project did not have the information on the new address of a participant, the participant’s information would be confirmed using the Basic Resident Register of each municipality. In viewing this information, we complied with the Basic Resident Registration Act. If a participant died, the TMM Project confirmed the death using the Basic Resident Register. After an application was submitted for outside use to confirm vital statistics, we applied to vital statistics to confirm cause of death.

#### Secondary survey

A secondary survey was conducted from 2017 (4 years after the baseline survey) to assess the changes in the health status of participants, including information about ophthalmic examination, hearing acuity, respiratory function tests, estimated central BP, calcaneal ultrasound bone mineral density, muscular strength, oral examinations, and other tests.

### Statistical analyses of baseline descriptive statistics

In this paper, we described information on current smoking and psychological distress assessed using the Kessler 6-item psychological distress scale.^19^ We compared age-adjusted prevalence of current smoking and higher K6 according to sex, recruitment methods, and residential area.

## RESULTS

### Baseline descriptive statistics

From May 2013, we identified 62,439 and 34,980 participants in the ToMMo and the IMM sites, respectively, to partake in the Type 1 surveys. In total 40,433 (65%) and 26,922 (77%) participants in the ToMMo and IMM provided informed consent to be enrolled. For the Type 1 Additional survey conducted only in the ToMMo, 664 participants provided informed consent to be enrolled. For the Type 2 surveys, 13,855 and 5,991 participants based in ToMMo or IMM provided informed consent to be enrolled, and 13,782 and 5,987 returned questionnaires, respectively (Table 4). More than 99% of participants provided blood and urine samples.

**Table 4.** Numbers of participants who were recruited for and participated in the TMM CommCohort study from 2013–2015 (fiscal years)

Recruiting results	ToMMo	IMM	Total
*Type 1 survey (28 municipalities in ToMMo and 18 municipalities in IMM)*			
Numbers who were recruited including “briefing” (A)	62,439	34,980	97,419
Numbers who participated (B+C+D)	40,433	26,922	67,355
Numbers who were withdrawal of consent as of March 31, 2018 (excluding disposal of all samples and information) (B)	636	248	884
Numbers who did not return questionnaire (C)	2,386	872	3,258
Numbers who returned questionnaire (D)	37,411	25,802	63,213
*Type 1 additional survey (5 municipalities in ToMMo)*			
Numbers who were recruited including “briefing” (E)	664	—	664
Numbers who participated (F+G+H)	664	—	664
Numbers who were withdrawal of consent as of March 31, 2018 (including disposal of all samples and information) (F)	4	—	4
Numbers who did not return questionnaire (G)	3	—	3
Numbers who returned questionnaire (H)	657	—	657
*Type 2 surveys*			
Numbers who were recruited including “briefing” (I)	13,855	5,991	19,846
Numbers who participated (J+K+L)	13,855	5,991	19,846
Numbers who were withdrawal of consent as of March 31, 2018 (including disposal of all samples and information) (J)	61	3	64
Numbers who did not return questionnaire (K)	12	1	13
Numbers who returned questionnaire (L)	13,782	5,987	19,769

*Detailed measurement*			
Numbers who participated in additional testing (I+M+N)	17,822	9,379	27,201
Numbers who recruited in Type 1 survey (M)	3,833	3,388	7,221
Numbers who recruited in Type 1 additional survey (N)	134	—	134
Numbers who participated in Type 2 surveys (I)	13,855	5,991	19,846

*All recruitment survey methods*			
Numbers who were recruited including “briefing” (A+E+I)	76,958	40,971	117,929
Numbers who participated (B+C+D+F+G+H+J+K+L)	54,952	32,913	87,865
Numbers who were withdrawal of consent as of March 31, 2018 (including disposal of all samples and information) (B+F+J)	701	251	952
Numbers who did not return questionnaire (C+G+K)	2,401	873	3,274
Numbers who returned questionnaire (D+H+L)	51,850	31,789	83,639

ToMMo, Miyagi Prefecture; IMM, Iwate Prefecture.

**Figure 3.**
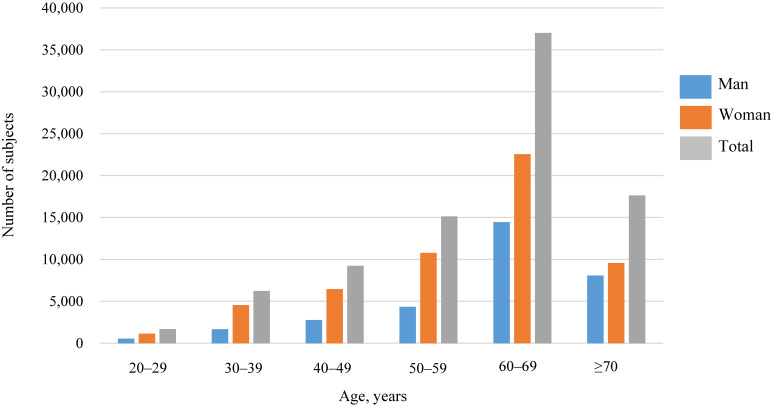
Age-sex distribution of participants who participated in the TMM CommCohort study from 2013–2015 (fiscal years) (*n* = 86,913)

Figure 3 shows the distribution of age groups in ToMMo and IMM. The mean age of participants who completed the Type 1 surveys, Type 1 Additional surveys, and the Type 2 surveys were 59.7, 50.5, and 57.4 years, respectively in ToMMo. Similarly, the mean age of participants who completed the Type 1 survey and Type 2 surveys in IMM were 61.8 and 54.7 years, respectively. Age- and sex-specific distributions of participants are provided in eTable 1.

**Figure 4.**
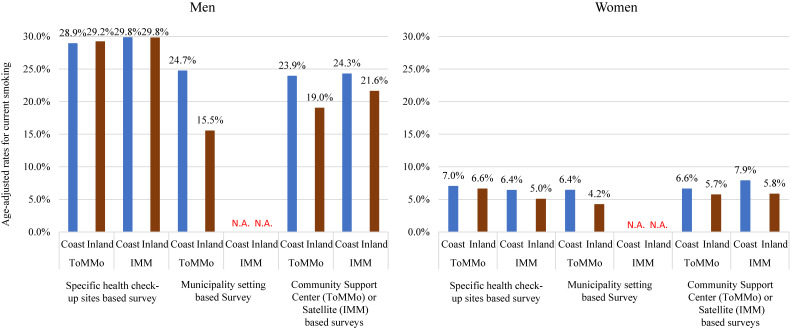
Age-adjusted rates for current smoking according to recruitment method/residents location in the TMM CommCohort study from 2013–2015 (fiscal years) (*n* = 82,427)

For the participants with Type 1 surveys, the prevalence of current smoking did not differ between coastal and inland areas (Figure 4). However, for volunteer-based recruitment, the prevalence of current smoking was higher in coastal areas than in inland areas. Except for IMM women, the prevalence of current smoking was higher in participants who completed the Type 1 surveys than those who completed the Type 2 surveys. Detailed information is provided in eTable 2.

**Figure 5.**
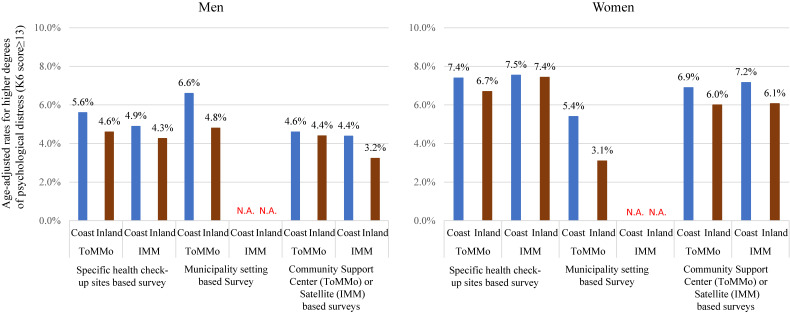
Age-adjusted rates for higher degrees of psychological distress (K6 score ≥13) according to recruitment method/residents location in the TMM CommCohort study from 2013–2015 (fiscal years) (*n* = 81,562)

The participants with Type 1 survey were more likely to have psychological distress assessed using the K6 than those of the Type 2 survey, and women were more likely to have psychological distress than the men (Figure 5). Additionally, coastal residents were more likely to have higher degrees of psychological distress than inland residents regardless of sex (the age-adjusted rates in men ranged from 3.2–4.8% and 4.4–6.6% in the inland and the coastal areas, respectively, and those in women ranged from 3.1–7.4% and 5.4–7.5% in inland and coastal areas, respectively). Detailed information is provided in eTable 3. Participation rate according to area was as follows: inland IMM, 68.9%; coastal IMM, 79.5%; inland ToMMo, 65.6%; and coastal ToMMo, 63.3%.

## DISCUSSION

### Large scale survey after natural disaster

The TMM CommCohort Study was a large scale prospective cohort study conducted in areas greatly impacted by the GEJE. Since we recruited participants from both coastal and inland areas, we could compare health status of participants between these areas. As shown in Figure 5, the prevalence of elevated K6 score was higher in coastal areas than that in inland areas. This difference may be because of housing damage and the death of relatives. Further analyses of this dataset will be performed in the future. We have a limitation in this topic. Some participants did not reside in Miyagi and Iwate Prefectures at the time of GEJE. This might cause inaccurate evaluation. However, the inclusion of these participants was unavoidable in a real-world setting. Additionally, this might not largely affect the main study findings.

### Higher consent rate in the Type 1 survey

As shown in Table 4, Type 1 survey based on municipal specific health check-up site achieved relatively higher consent rate; around 70% of participants who visited health check-up participated in our study. Because we asked participants to provide their genomic information, it might be unrealistic to obtain the very high consent rate for this survey. We considered our consent rate was satisfactorily high. Of course, participants who undergo health check-ups have higher health consciousness than those who do not,^23^ and participants who agreed to participate in our study might have higher health consciousness. Thus, although we considered that our participants satisfactory represent the participants who underwent health check-ups in Miyagi and Iwate areas, we should take care that the prevalence obtained from our survey might underestimate the prevalence in all participants. However, this effect of health consciousness might be almost identical among all participants in this survey; we considered that the internal validity of our participants was preserved.

### Detailed measurement in the Type 2 survey

Conversely, the Type 2 survey recruited participants who voluntarily visited our assessment center. Thus, these participants might not represent the general population who lived in Miyagi and Iwate Prefectures. However, the surveys provide a lot of detailed information on health status, as shown in Table 3A and Table 3B. This information might contribute to understanding the effect of disaster-related factors on health status. Furthermore, following up these detailed health conditions might clarify not only long-term effects of the natural disaster on health status but also general issues that affect atherosclerosis progression, worsening pulmonary function, and other diseases. As with the Type 1 survey, since all participants voluntarily participated in this survey, internal validity was preserved. However, these volunteers might have higher health consciousness. In fact, smoking status was different from the Type 1 and Type 2 surveys, as shown in Figure 4. Thus, there was a problem in simply analyzing the combination of recruitment methods; an analysis, such as a stratified analysis, should be required to handle these data simultaneously.

### Genome and omics information

We asked participants to provide their genomic information, and we have already analyzed whole genome and array information. As of March 2019, most of genome information has been analyzed in this cohort. Thus, our information can be analyzed together with genome information. We have already reported the several papers regarding genome-wide association analysis or gene-environment interaction for health status.^24^^,^^25^ Furthermore, we also analyzed information on metabolome.^26^ Thus, we could analyze the gene-metabolome association, and the metabolome-disease association. In addition, we considered that using metabolome information might clarify the pathway from gene to health status. We have also added epigenomic information to this cohort,^27^ and we conducted epigenome-wide association studies.^28^

### As a biobank

In this project, we have obtained the agreement of all the participants to transfer their biological samples and data to researchers. Although only a part of our cohort information has already been distributed, several groups used our information as controls, and several collaborative studies were published.^29^^–^^34^ We will expand the data size and will add the follow-up information, including mail surveys; information on medical expenditures and health check-up; medical record review; public statistics, such as basic resident registration and vital statistics; and secondary survey data. This expansion may contribute to the development of personalized medicine and healthcare.

### Data distribution and collaborative study

Collaborations are highly welcome. Please contact the corresponding authors (AH, KT) with any enquiries. We also established a system for data transfer to Japanese researchers. Information on data distribution is provided on the webpage (http://www.dist.megabank.tohoku.ac.jp/ currently in Japanese only).

### Conclusion

This cohort comprised a large sample size and contains information on the natural disaster, genome information, and metabolome information. This cohort also had several detailed measurements. Using this cohort enabled us to clarify the long-term effect of the natural disaster and also to establish personalized prevention based on genome, metabolome, and other omics information.

## APPENDIX A. SUPPLEMENTARY DATA

eTable 1. Age-sex distribution of participants who participated in the TMM CommCohort study from 2013–2015 (fiscal years) (*n* = 86,913)

eTable 2. Age-adjusted prevalence for current smoking in the TMM CommCohort study from 2013–2015 (fiscal years) (*n* = 82,427)

eTable 3. Age-adjusted rates for higher degrees of psychological distress (K6 score ≥13) in the TMM CommCohort Study from 2013–2015 (fiscal years) (*n* = 81,562)
