# Supplementary material for: Study Profile of the Tohoku Medical Megabank Community-Based Cohort Study
Source: J Epidemiol. 2021 Jan 5;31(1):65–76. doi: 10.2188/jea.JE20190271 (PMC7738642; doi:10.2188/jea.JE20190271)
Supplement: Supplementary file 1 [file je-31-065-s001.pdf]

eTable 1. Age-sex distribution of participants who participated in the TMM CommCohort study from 2013–2015 (fiscal years)(n=86,913)

Age at baseline survey, years		20–29	30–39	40–49	50–59	60–69	≥70	Total		Mean age (SD)	
Specific health check-up sites based survey											
ToMMo	Men	229	751	1,167	1,940	7,593	3,735	15,415	39,797	61.8 (10.8)	59.7 (11.6)
	Women	427	2,196	2,629	4,564	10,804	3,762	24,382		58.4 (11.8)	
IMM	Men	75	322	820	1,343	4,602	2,821	9,983	26,674	62.8 (10.0)	61.8 (10.4)
	Women	139	816	1,504	2,867	7,507	3,858	16,691		61.2 (10.5)	
Municipality setting based survey											
ToMMo	Men	20	66	47	34	84	32	283	660	51.2 (15.2)	50.5 (14.4)
	Women	29	70	79	84	92	23	377		50.0 (13.8)	
IMM	Men	-	-	-	-	-	-	-	-	-	-
	Women	-	-	-	-	-	-	-		-	
Community Support Center (ToMMo, Miyagi) or Satellite (IMM, Iwate) based surveys											
ToMMo	Men	134	349	431	578	1,534	1,101	4,127	13,794	60.2 (14.0)	57.4 (13.8)
	Women	371	994	1,470	2,143	3,207	1,482	9,667		56.2 (13.6)	
IMM	Men	88	194	298	449	631	376	2,036	5,988	57.0 (14.4)	54.7 (13.9)
	Women	180	475	785	1,125	945	442	3,952		53.6 (13.4)	
All recruitment methods											
ToMMo	Men	383	1,166	1,645	2,552	9,211	4,868	19,825	54,251	61.3 (11.7)	59.0 (12.3)
	Women	827	3,260	4,178	6,791	14,103	5,267	34,426		57.7 (12.4)	
IMM	Men	163	516	1,118	1,792	5,233	3,197	12,019	32,662	61.8 (11.1)	60.5 (11.4)
	Women	319	1,291	2,289	3,992	8,452	4,300	20,643		59.7 (11.6)	
Total	Men	546	1,682	2,763	4,344	14,444	8,065	31,844	86,913	61.5 (11.5)	59.6 (12.0)
	Women	1,146	4,551	6,467	10,783	22,555	9,567	55,069		58.5 (12.1)	
	Men + Women	1,692	6,233	9,230	15,127	36,999	17,632	86,913	-	-	-

The number of participants excluded those who withdrew consent (disposal of any samples and information) until 31st March 2018 (ToMMo: n = 54,251, IMM: n=32,662, Total: n=86,913).

ToMMo, Miyagi Prefecture; IMM, Iwate Prefecture; SD, standard deviation.

eTable 2. Age-adjusted prevalence for current smoking in the TMM CommCohort study from 2013–2015 (fiscal years)(n=82,427)

		Specific health check-up sites based survey				Municipality setting based survey				Community Support Center (ToMMo) or Satellite (IMM) based surveys			
		ToMMo		IMM		ToMMo		IMM		ToMMo		IMM	
Age classes	Standard population	Coast	Inland	Coast	Inland	Coast	Inland	Coast	Inland	Coast	Inland	Coast	Inland
Men													
20–29	1,692	26.0%	35.6%	25.4%	36.4%	20.0%	13.3%	-	-	40.0%	36.5%	41.9%	34.6%
30–39	6,233	51.0%	44.7%	46.4%	32.9%	31.6%	34.8%	-	-	44.4%	38.4%	42.5%	31.3%
40–49	9,230	45.6%	44.2%	42.1%	44.4%	10.5%	21.4%	-	-	41.2%	26.0%	42.9%	29.4%
50–59	15,127	36.3%	40.5%	41.2%	43.6%	50.0%	25.9%	-	-	30.8%	27.1%	33.9%	32.8%
60–69	36,999	24.5%	24.8%	26.4%	27.3%	20.0%	14.1%	-	-	19.2%	16.1%	20.0%	21.5%
≥70	17,632	15.7%	14.6%	15.4%	13.8%	18.2%	0.0%	-	-	9.9%	6.3%	7.1%	3.4%
Age-adjusted rates*		28.9%	29.2%	29.8%	29.8%	24.7%	15.5%	-	-	23.9%	19.0%	24.3%	21.6%
Women													
20–29	1,692	15.2%	15.2%	17.1%	16.7%	0.0%	8.0%	-	-	13.4%	15.9%	17.7%	9.1%
30–39	6,233	15.3%	15.5%	16.4%	13.3%	17.9%	11.9%	-	-	14.8%	13.1%	15.5%	11.9%
40–49	9,230	15.8%	14.8%	16.2%	11.0%	15.0%	11.9%	-	-	14.8%	12.2%	18.1%	15.6%
50–59	15,127	10.8%	9.5%	8.3%	7.2%	9.5%	11.1%	-	-	9.0%	6.4%	9.7%	6.7%
60–69	36,999	3.6%	3.7%	3.3%	2.8%	4.3%	0.0%	-	-	4.2%	3.9%	5.3%	4.0%
≥70	17,632	2.4%	1.8%	1.6%	0.7%	0.0%	0.0%	-	-	1.8%	1.8%	2.6%	1.2%
Age-adjusted rates*		7.0%	6.6%	6.4%	5.0%	6.4%	4.2%	-	-	6.6%	5.7%	7.9%	5.8%

*The age-adjusted rates for current smoking were calculated using the direct method of standardization, i.e., Σ(ri*ni)/N.

Here, ri is the i-th age-group specific rate for current smoking, ni is the observations of the i-th age group in the standard population, and N is the total number of observations in the standard population (N = Σni).

The age-adjusted rates were calculated for each of subgroups, defined by sex and type of recruitment, and the entire TMM CommCohort population itself was used as the standard population.

Analytic subjects (ToMMo: n = 51,079, IMM: n = 31,348) were included to classify 3 categories of smoking (current smoking, past smoking, or never smoking) from response to the smoking questions.

ToMMo, Miyagi Prefecture; IMM, Iwate Prefecture.

eTable 3. Age-adjusted rates for higher degrees of psychological distress (K6 score≥13) in the TMM CommCohort Study from 2013–2015 (fiscal years)(n=81,562)

		Specific health check-up sites based survey				Municipality setting based survey				Community Support Center (ToMMo) or Satellite (IMM) based surveys			
		ToMMo		IMM		ToMMo		IMM		ToMMo		IMM	
Age classes	Standard population	Coast	Inland	Coast	Inland	Coast	Inland	Coast	Inland	Coast	Inland	Coast	Inland
Men													
20–29	1,692	15.7%	14.6%	6.9%	9.1%	20.0%	13.3%	-	-	13.8%	24.3%	14.5%	12.0%
30–39	6,233	17.6%	12.6%	10.4%	14.7%	15.8%	2.2%	-	-	9.0%	13.9%	12.7%	7.5%
40–49	9,230	11.7%	11.3%	9.6%	7.0%	10.5%	10.7%	-	-	5.7%	9.4%	7.1%	4.9%
50–59	15,127	7.4%	4.7%	6.6%	4.2%	0.0%	7.4%	-	-	8.0%	6.0%	5.9%	2.6%
60–69	36,999	2.1%	2.3%	3.1%	2.3%	5.0%	4.7%	-	-	2.9%	1.4%	2.3%	1.8%
≥70	17,632	2.9%	1.9%	2.6%	3.0%	9.1%	0.0%	-	-	2.3%	1.6%	2.1%	3.5%
Age-adjusted rates*		5.6%	4.6%	4.9%	4.3%	6.6%	4.8%	-	-	4.6%	4.4%	4.4%	3.2%
Women													
20–29	1,692	17.7%	18.3%	20.2%	16.7%	33.3%	32.0%	-	-	17.3%	19.8%	21.2%	18.2%
30–39	6,233	11.7%	11.0%	14.0%	15.6%	28.6%	7.1%	-	-	15.6%	12.1%	13.4%	10.1%
40–49	9,230	11.0%	11.8%	11.4%	15.1%	0.0%	5.1%	-	-	11.1%	9.4%	10.5%	7.4%
50–59	15,127	9.9%	8.9%	8.9%	8.3%	4.8%	4.8%	-	-	7.2%	7.7%	6.8%	8.0%
60–69	36,999	5.6%	4.7%	5.7%	4.0%	4.3%	1.5%	-	-	5.0%	3.9%	4.8%	4.3%
≥70	17,632	4.8%	3.7%	4.8%	6.1%	0.0%	0.0%	-	-	4.5%	3.5%	7.1%	4.8%
Age-adjusted rates*		7.4%	6.7%	7.5%	7.4%	5.4%	3.1%	-	-	6.9%	6.0%	7.2%	6.1%

*The age-adjusted rates for higher degrees of psychological distress (K6 score≥13) were calculated using the direct method of standardization, i.e., Σ(ri*ni)/N.

Here, ri is the i-th age-group specific rate for higher degrees of psychological distress, ni is the observations of the i-th age group in the standard population, and N is the total number of observations in the standard population (N = Σni).

The age-adjusted rates were calculated for each of subgroups, defined by sex and type of recruitment, and the entire TMM CommCohort population itself was used as the standard population.

Analytic subjects (ToMMo: n = 50,679, IMM: n = 30,883) were included to classify 2 categories of the presence of psychological distress (K6 score<12 or ≥13) from response to the K6 questions.

ToMMo, Miyagi Prefecture; IMM, Iwate Prefecture.
